# Multisystem Inflammatory Syndrome in Children (MIS-C) Following SARS-CoV-2 Infection: Role of Oxidative Stress

**DOI:** 10.3389/fimmu.2021.723654

**Published:** 2021-10-19

**Authors:** Omar Graciano-Machuca, Geannyne Villegas-Rivera, Iván López-Pérez, José Macías-Barragán, Sonia Sifuentes-Franco

**Affiliations:** ^1^ Laboratory of Biological Systems, Department of Health Sciences, University of Guadalajara (UDG), Ameca, Mexico; ^2^ Department of Health Sciences—Disease as an Individual Process, University of Guadalajara (UDG), Tonalá, Mexico

**Keywords:** COVID-19, oxidative stress, Multisystem Inflammatory Syndrome in Children, inflammation, cytokines

## Abstract

With the appearance of the SARS-CoV-2 virus in December 2019, all countries in the world have implemented different strategies to prevent its spread and to intensively search for effective treatments. Initially, severe cases of the disease were considered in adult patients; however, cases of older school-age children and adolescents who presented fever, hypotension, severe abdominal pain and cardiac dysfunction, positive for SARS-CoV-2 infection, have been reported, with increased pro-inflammatory cytokines and tissue damage, condition denominated multisystemic inflammatory syndrome (MIS-C); The emerging data from patients with MIS-C have suggested unique characteristics in the immunological response and also clinical similarities with other inflammatory syndromes, which can support as a reference in the search for molecular mechanisms involved in MIS-C. We here in propose that oxidative stress (OE) may play a very important role in the pathophysiology of MIS-C, such as occurs in Kawasaki disease (KD), severe COVID-19 in adults and other processes with characteristics of vascular damage similar to MIS- C, for which we review the available information that can be correlated with possible redox mechanisms.

## Introduction

In December 2019, with the recent emergence of the new coronavirus, severe acute respiratory syndrome coronavirus 2 (SARS-CoV-2) has started to appear in China (1). Its subsequent spread globally has forced drastic lifestyle changes around the world after being declared a pandemic by the World Health Organisation (WHO) (2). This virus causes a complicated infectious disease called coronavirus disease 2019 (COVID-19) (3), which has become a global health threat. Until August 2021, COVID‐19 has caused the death of 4,517, 240 individuals worldwide, with more than 217,000,000 cumulative confirmed cases (2).

## SARS-CoV-2 and COVID-19

### SARS-CoV-2

Coronaviruses (CoVs) are a family of enveloped ribonucleic acid (RNA) viruses that cause infections that are transmitted mainly through the respiratory and fecal-oral routes. This family of viruses is distinguished by the large size of its genome (4); CoVs are classified into four genera based on phylogeny: group 1 or alpha-CoV, group 2 or beta-CoV, group 3 or gamma-CoV and group 4 or delta-CoV (5).

The SARS-CoV-2 is a recently identified beta-coronavirus with a size genome of 29.9 kb, encoding at least 29 proteins, four of which are structural proteins: the spike (S), membrane (M), envelope (E) and nucleocapsid (N) proteins (6, 7).

### SARS-CoV-2 Infection and Clinical Aspects of COVID-19

**Figure 1:**
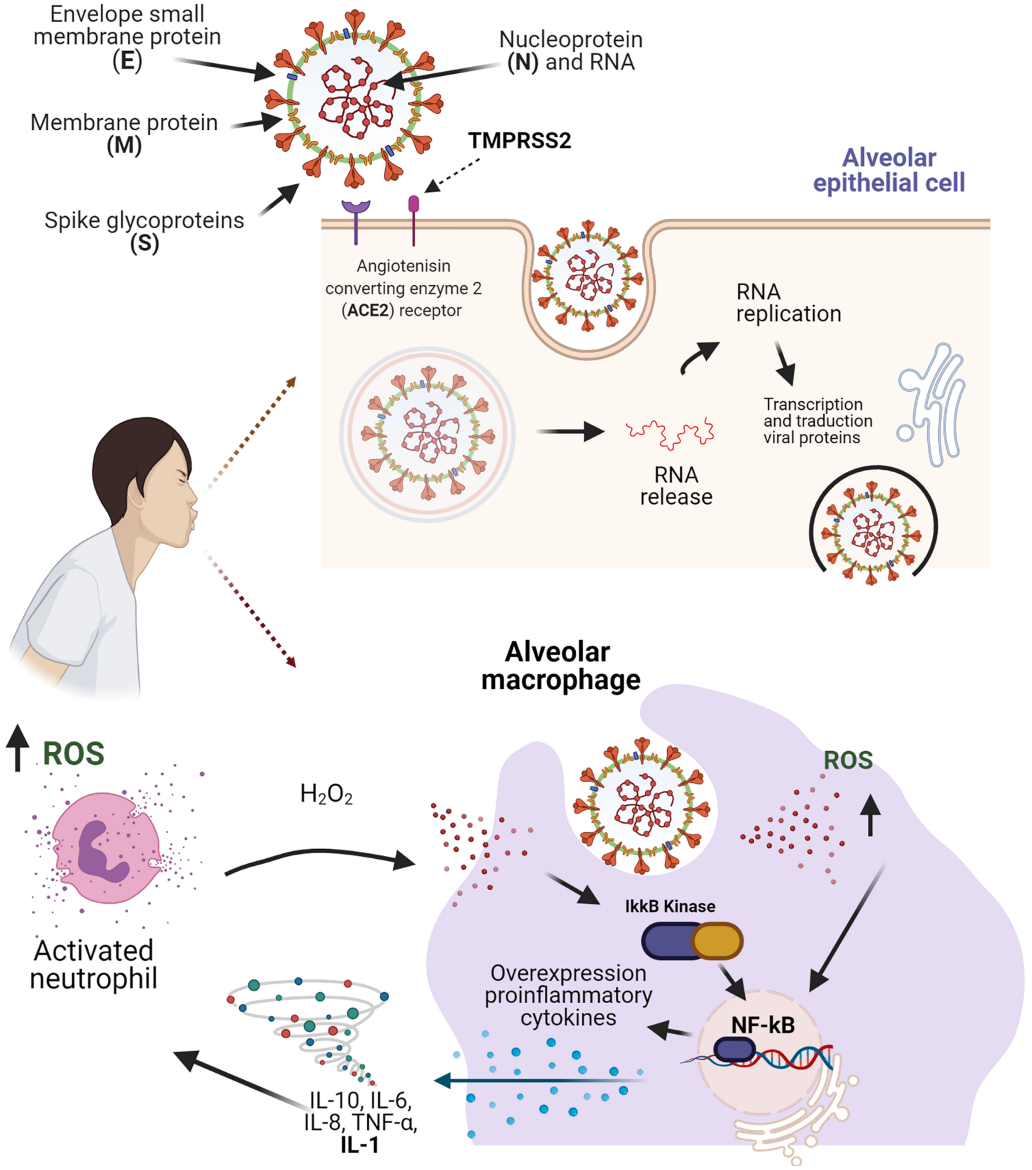
Structure of the SARS-CoV-2 virus and molecular mechanisms in the inflammation process and oxidative stress. The binding of the S protein to a receptor ACER2 and the co-receptor TMPRSS2, allows the SARS-CoV-2 entry the alveolar epithelial cell. The recognition of SARS-CoV-2 in the macrophages, leads the accumulation of ROS and facilitates the activation of the NF-kB pathway, which results in an expression of pro-inflammatory cytokines that promote neutrophil activation. Figure created with BioRender, ©biorender.com.

The SARS-CoV-2 is transmitted by droplets generated during coughing and sneezing by infected people without adequate personal protection and hygiene, regardless of whether they are symptomatic or not (8, 9). In SARS-CoV-2 infection, the virus enters the host cells using the S protein through an interaction with host cell receptors (ACE2) and the processing of S protein by endogenous transmembrane serine protease 2 (TMPRSS2), leading to endocytosis and the release of viral RNA, which is replicated and translated into viral proteins to facilitate viral replication (8) (**Figure 1**).

The severity is highly variable among individuals infected with SARS-CoV-2, ranging from asymptomatic cases up to severe respiratory disease with extrapulmonary findings (10–12).

Initially, people over 65 years of age represented a population at high risk of complications, with approximately 50% of hospitalizations and up to 80% of deaths caused by COVID-19 (13), whereas the pediatric population was considered less affected in frequency and severity (14). However, in late April 2020, the first reports of a childhood multisystem inflammatory condition associated to SARS-CoV-2 infection were published in countries such as the United Kingdom, Italy and the United States (US) (15, 16). “Inflammatory Multisystem Syndrome Temporally Associated with SARS-CoV-2 Infection” (PIMS-TS) in Europe and Multisystem Inflammatory Syndrome in Children (MIS-C) in the US (17).

## MIS-C

The MIS-C is a systemic inflammation that comprises the heart, lungs, kidneys, brain, skin, eyes and gastrointestinal organs; the diagnostic criteria are shown in **Table 1**. The onset of symptoms begins at least 2 weeks after SARS-CoV2 infection (17); some are shared with other pathological entities such as toxic shock syndrome (TSS), macrophage activation syndrome (MAS) and Kawasaki disease (KD) (20, 21). Therefore, the clinical manifestations, the role of SARS-CoV-2 infection and the pathophysiological mechanisms of MIS-C are still unknown. In this sense, it is still uncertain whether the symptoms are due to post-infectious hyper-inflammation, autoinflammatory or autoimmune disease (17, 21, 22).

**Table 1:** Diagnostic criteria for PIMS-TS/MIS-C.

World Health Organization (2)	Royal College of Pediatrics and Child Health (UK) (18)	Centers for Disease Control and Prevention (US) (19)
1. 0–19 years of age. 2. Fever > 3 days and 2 of the following: a) Rash or bilateral non-purulent conjunctivitis or muco-cutaneous inflammation signs (oral, hands or feet). b) Hypotension or shock. c) Features of myocardial dysfunction, pericarditis, valvulitis, or coronary abnormalities (including ECHO findings or elevated Troponin/NT-proBNP), d) Evidence of coagulopathy (by PT, PTT, elevated D-dimer). e) Acute gastrointestinal problems (diarrhea, vomiting, or abdominal pain). 3. Elevated markers of inflammation such as ESR, C-reactive protein, or procalcitonin. 4. No other obvious microbial cause of inflammation, including bacterial sepsis, staphylococcal or streptococcal shock syndromes. 5. Evidence of COVID-19 (RT-PCR, antigen test or serology positive), or likely contact with patients with COVID-19.	1. Pediatric age group. 2. Persistent fever > 38.5°C. 3. Evidence of single or multi-organ dysfunction (shock, cardiac, respiratory, renal, gastrointestinal or neurological disorder) with additional features, which may include children fulfilling full or partial criteria for KD. 4. Inflammation markers (neutrophilia, elevated CRP and lymphopenia). 5. SARS-CoV-2 PCR testing may be positive or negative.	1. An individual aged <21 years. 2. Fever ≥38.0°C for ≥24 hours, or report of subjective fever lasting ≥24 hours. 3. Laboratory evidence of inflammation: an elevated CRP, ESR, fibrinogen, procalcitonin, D-dimer, ferritin, LDH, or IL-6, elevated neutrophils, reduced lymphocytes and low albumin. 4. Severe illness necessitating hospitalization. 5. 2 or more organ systems affected (e.g., cardiac, renal, respiratory, hematologic, gastrointestinal, dermatologic, and neurological. 6. Positive for current or recent SARS-CoV-2 infection by RT-PCR, serology, or antigen test; or COVID-19 exposure within the 4 weeks prior to onset of symptoms.

NT-proBNP, N-terminal pro-brain natriuretic peptide; PT, prothrombin; PTT, partial thromboplastin time; KD, Kawasaki disease; CRP, C-reactive protein; ESR, erythrocyte sedimentation rate; IL, interleukin; LDH, lactate dehydrogenase; RT-PCR, reverse transcription-polymerase chain reaction.

Laboratory findings include lymphopenia and reduced to normal thrombocytes (21). In the acute phase, there is an increase in inflammatory cytokines and other molecules, such as interleukin-1β (IL-1β), IL-6, IL-8, tumor necrosis factor-α (TNF-α), IL-10, IL-17, interferon-γ (IFN-γ), IL-2 receptor agonist, C-reactive protein (CRP) and ferritin. Additionally, markers of myocardial dysfunction and injury are also increased, including N-terminal pro B-type natriuretic peptide (NT-proBNP) and troponin. In contrast to KD, in MIS-C, there is evidence of a procoagulant state establishment due to raised fibrinogen and D-dimer levels and low platelet count (17).

During the acute phase, there is lymphopenia of helper (CD4^+^), cytotoxic (CD8^+^) and γδ T cells. The count of total neutrophils, monocyte, dendritic cell and natural killer cells remains normal, despite the expression of molecules associated with their function changes. High levels of IL-8 could be related to increased neutrophil activation, which in turn could influence T and B lymphocyte responses. In contrast, molecules involved in antigen presentation have decreased expression on macrophages and dendritic cells, suggesting impaired function (23).

## Oxidative Stress and COVID-19

The OS is an imbalance between the production of RS and the endogenous antioxidant defense, indicating an imbalance of the pro-oxidant-antioxidant balance in favor of pro-oxidants (24). Two types of reactive species are distinguished: reactive oxygen species (ROS) and reactive nitrogen species (RNS) (25) both types of RS are highly reactive, and their interaction with macromolecules can lead to permanent modifications and altered cell signaling events (26).

OS has been implicated in different pathologies as a triggering factor of the pathophysiological process, such as kidney disease (27), diabetes (28), rheumatic autoimmune disease (24, 29) In addition to its role in these chronic diseases, the production of ROS plays an important role in the defense mechanisms against infectious diseases such as hepatitis B, C and D, herpes, influenza, tuberculosis and leprosy (30–33). However, excessive ROS production increases the inflammatory response and in turn can increase cellular injury induced by viral infection (34). Evidence suggests that OS is involved in acute bronchiolitis caused by respiratory syncytial virus, considering elevated plasma levels of oxidized glutathione (GSSG) and the antioxidant enzyme glutathione peroxidase (GPx) as biomarkers of severity in these patients (35).

Most viral infections, after the invasion of host tissues, hijack cellular functions to initiate viral replication; however, the immune system can limit viral spread through the recognition of innate elements in the infected cells, followed by the activation of defense mechanisms to eradicate the infection *via* the involvement of cells such as macrophages, epithelial cells, dendritic cells and their pattern recognition receptors (36). In SARS-CoV2 infection, in addition to the participation of these cells, it has been described that mast cells (MC) play a fundamental role in inflammation and severity of patients, through the release of histamine by MC caused by the recognition of the virus and subsequent IL-1 secretion (37).

Some of these pathways increase the activity of type I IFN and inflammatory cytokines (38). After viral replication, there is an increase in the levels of TNF-α, IFN-γ and IL-4, which leads to the activation of cellular pro-inflammatory pathways (39). Importantly, in addition to promoting cytotoxicity, ROS and RNS can initiate and amplify inflammatory pathways through the regulation of gene expression *via* nuclear factor kappa B (NF-κB) (40). Moreover, the presence of ROS, such as hydrogen peroxide (H_2_O_2_), usually functions as a stimulus for the activation of inflammatory responses (41, 42).

The NF-κB pathway is essential to regulate multiple cellular processes, especially inflammatory responses, apoptosis and the differentiation of immune system cells. Several factors can activate the NF-κB pathway, for example TNFα, lipopolysaccharide (LPS) and IL-1 (43). The activation of this transcriptional factor during viral infections has been previously evaluated, demonstrating its role in processes such as promoting viral replication and regulating the host immune response (44). The hyperactivation of this transcription factor is an important key for the progression of severe cases of COVID-19, since in these critically ill patients, the levels of pro-inflammatory cytokines and ROS are significantly higher (45).

In severe cases of COVID-19, there is a significant decrease in oxygen saturation (less than 90%), which produces significant hypoxic changes (46, 47). This events also lead to the development of OS just as the reduction in oxygen saturation enhances the formation of ROS such as superoxide $\left({\text{O}}_{2}^{-}\right)$ and peroxide (H_2_O_2_) that, in turn, cause changes in membrane lipids, nucleic acids and other cellular structures (48). For this reason, this pathway NF-κB could be a key element to understand the role of OS in severe cases of COVID-19 through increasing the inflammatory process (49). Likewise, it is known that during SARS-Cov-2 infection, local and systemic inflammation is generated mediated by pro-inflammatory cytokines, mainly by IL-1, a cytokine with a wide spectrum of biological activities: activation of adhesion molecules, endothelial dysfunction, stimulation in the secretion of TNF and IL-6, as well as an increase in nitric oxide and the release of prostaglandins and thromboxane A2 (50), the latter highly related to the development of OS and endothelial damage. On the other hand, it has been proposed that the suppression of the IL-1 and IL-6 response could have therapeutic effects in the management of patients with COVID-19, through an approach directed at IL-37 and IL-38 (51).

Another pathway that correlates OS with SARS-CoV2 infection is the Nrf-2 factor (nuclear factor erythroid-derived 2-related factor 2), which regulates approximately 250 genes involved in cell homeostasis, including antioxidant proteins and numerous cytoprotective proteins (52). The role of Nrf2 in inflammation, immunity and aging is well known, and it has also been shown that the severity of COVID-19 infection is inversely associated with the expression of Nrf2 (53). *S*tudies have shown that the use of Nrf2 activators suppresses the inflammatory response and generates antiviral effects that can help reduce viral replication, improving the prognosis of patients with hyper-inflammation with COVID-19 (54, 55).

Both NF-κB and Nrf2 are regulated by oxidative factors: the blocking of Nrf2 is associated with increased oxidative and nitrosative stress, leading to amplification of cytokine production, and the NF-κB factor is readily activated in oxidative environments (56) (**Figure 1**). For this reason, some authors consider that the Nrf2 and NF-κB pathways are involved in the development and progress of inflammatory pathology in COVID-19, and the different treatments aimed at this signaling pathway offer a tool in the management of the patients (57).

The involvement of OS in the progression and severity of COVID-19 has not been fully demonstrated; however, a study showed the increase of high-sensitivity CRP (hs-CRP), a marker of OS, in patients that died after COVID-19 disease (58). The administration of antioxidants supports the hypothesis that the re-establishment of redox conditions in cells could be a suitable approach for the concomitant treatment of multiple diseases associated with OS. Moreover, recent studies have shown that vitamin D supplementation, as adjuvant therapy, improves the prognosis of patients with COVID-19; in some cases supplemented with vitamin D and B_12_, oxygen requirements decreased compared with the non-supplemented group (58, 59).

### Influence of OS on the Pathophysiology of MIS-C

The pathophysiology of MIS-C is not exactly known, and OS could play an important role: first, OS regulates the NF-κB, which in turn regulates the immune response and inflammation in several viral infections (17, 20). Second, previous studies have shown that OS is involved in the acute stage of KD, caused by an overproduction of ROS for activated inflammatory cells (60). Although MIS-C has clinical similarities and cytokine profiles comparable to KD and TSS, recent studies suggest that there are differences in the type of activated cells during the immune response, observing a specific expansion of activated T lymphocytes that express the Vβ21.3 T cell receptor β chain variable region in CD4 and CD8 cells, something that differs in KD, TSS patients or SARS-COV-2 (61). In addition, the antibody response in MIS-C compared to adults with severe COVID-19 presents important differences, for example, a study showed that patients with MIS-C produced predominantly specific IgG antibodies (Abs) for the S protein but not for the N protein and in addition to a reduced neutralizing activity, while adult patients with COVID-19 presented Abs anti-S IgG, IgM and IgA, as well as Abs anti-N IgG, these results suggested that the immunological profile in MIS-C presents certain differential characteristics of COVID-19 in adults (62). Similarly, it has been reported that patients with MIS-C present an immunological profile that is characterized by strong activation of CX3CR1 + CD8 + T cells of vascular patrolling however this activation decreases with time and is correlated with clinical improvement of patients, which only occurs in patients with MIS-C and not in pediatric patients with COVID-19 without MIS-C (63). Despite these specific differences between COVID-19 in adults, pediatric patients without MIS-C versus MIS-C, there are similar immunological characteristics between MIS-C and severely ill adult patients with COVID-19, in which it has been shown that the redox state is strongly altered, observing an increase in lipid peroxidation, and a deficit of some antioxidants (vitamin C, glutathione), a situation that could be similar in patients with MIS-C (64).

**Figure 2:**
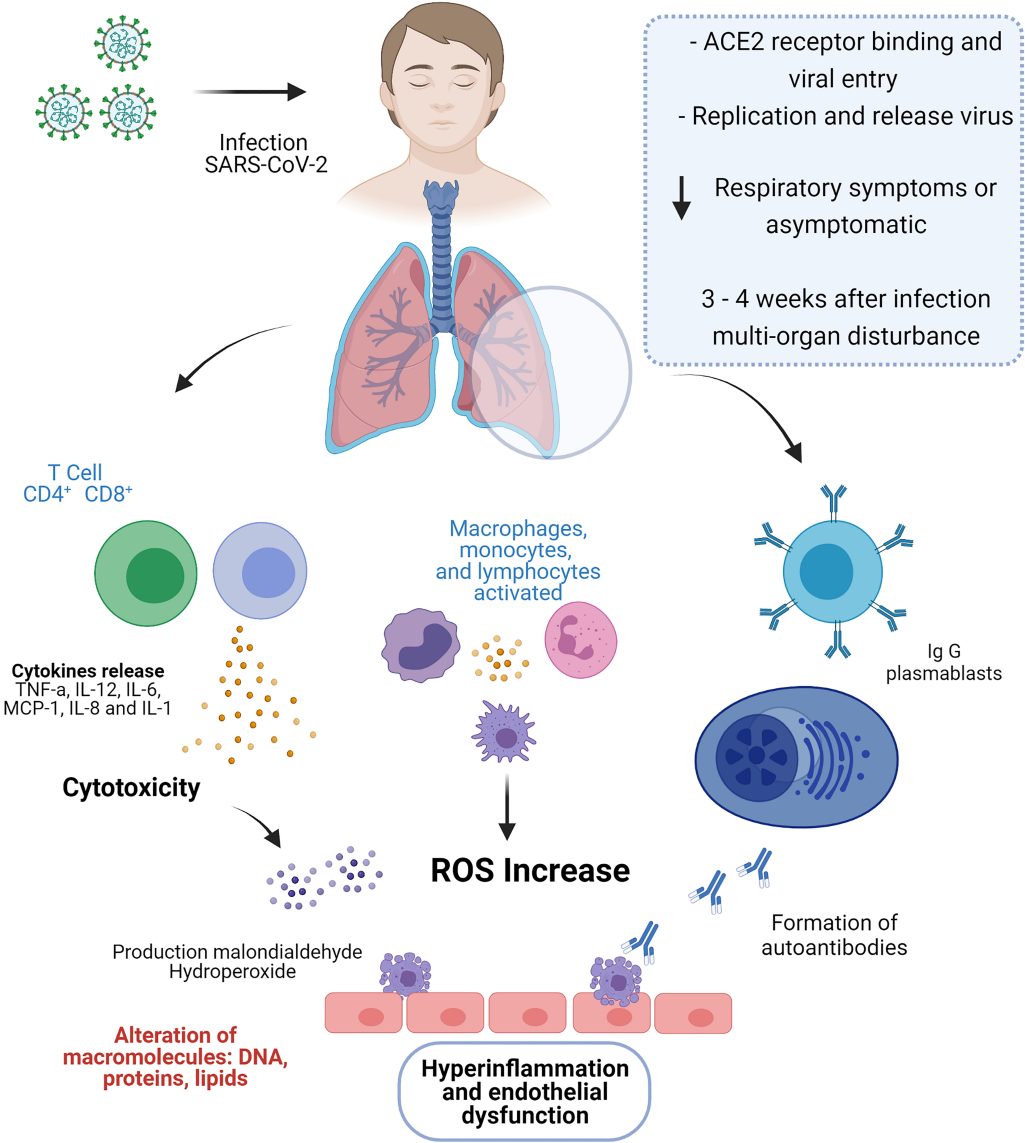
In SARS-CoV-2 infection, the binding of the receptor ACE2 with the virus leads to entry into the host cell and its subsequent replication and release. During the active phase of COVID-19, MIS-C patients were asymptomatic or with mild respiratory symptoms; however, three to four weeks after exposure to SARS-CoV-2, children develop a multi-organ disturbance. MIS-C is characterized by strong activation of T lymphocytes leading to the release of a cytokine storm and increased cytotoxic activity. Furthermore, T cells, together with monocytes and macrophages, promote oxidative stress by increasing reactive oxygen species, which alter macromolecules such as DNA, proteins and lipids. Additionally, the presence of autoantibodies that recognize endothelial cells, promoting damage and contributing to endothelial dysfunction and multisystem inflammation typical of MIS-C. Figure created with BioRender, ©biorender.com.

The pathophysiology of COVID-19 in adults includes endothelitis and lymphocytic endothelitis in organs such as the lung, heart, kidney and liver (65), this endothelial dysfunction has been associated with an increase in the hormone that regulates vascular tone (endthelin-1) and changes in the coagulation; despite the differences in the immune response between COVID-19 in adults and MIS-C (previously mentioned), there are similar events such as endothelitis, which have been proposed to describe the pathophysiology in MIS-C, identifying the presence of IgG and IgA autoantibodies that recognize endothelial cells, promoting damage and contributing to endothelial dysfunction and multisystem inflammation typical of MIS-C (66, 67). In patients with atherosclerosis, hypertension, endotoxic shock or infectious diseases, an increase in endothelin-1 has been described, which is attributed pro-inflammatory activities, platelet aggregation and also plays a role in the increase in the expression of adhesion molecules of leukocytes (68), causing endothelial cell injury; this endothelial dysfunction is also consistent and is related to mortality in patients with COVID-19 (69), in severe patients with MIS-C endothelial dysfunction could explain its clinical presentation (**Figure 2**).

As mentioned previously, MIS-C has been associated due to its clinical characteristics with other inflammatory syndromes (TSS and KD), for which some common pathways and divergences are mentioned that may explain the participation of OS in MIS-C. It has been proposed that platelet count can help differentiate KD and MIS-C, since KD patients are characterized by normal/high platelet counts while MIS-C patients commonly present with lower platelet counts (70) which leads us to reinforce our hypothesis that OS could be involved in this process, as occurs in immune thrombocytopenia (IT), in which multiple lines of evidence suggest that OS participates in the pathogenesis of this disorder, observing elevation of lipid peroxidation and the reduction of antioxidant capacity, as well as the use of antioxidants improves the prognosis in these patients (71, 72). It has also been described that patients with acute MIS-C present increased binding of antibodies to antigens associated with the development of the endothelium and the heart and other common targets with autoimmunity compared to healthy controls (73). Likewise, the possibility is raised that in patients with severe MIS-C, there is the formation of autoantibodies, which bind to endothelial cells, which contribute to endothelial dysfunction and multisystem inflammation characteristic of these patients (74). Although there is no unifying hypothesis on how ET-1 affects this process in patients with MIS-C, its significant role in the progression of the pathology is not ruled out, such as occurs in KD where the levels of immunoreactive endothelin (iET) in the blood are increased as a result of associated vascular endothelial damage (75). The biosynthesis and release of ET-1 are regulated at the transcriptional level by different factors such as the kinase p38MAP, NF-κB, PKC/ERK and JNK/c-Jun, which in turn are activated in the presence of OS (76), which may reinforce the possible participation of OS in the pathogenesis of MIS-C, considering the similarities with other diseases with a characteristic of endothelial damage.

It is evident that in inflammatory syndromes there is an exacerbated and uncontrolled immune response, such as occurs with MIS-C; Therefore, prior knowledge of these syndromes can support the description of the pathophysiology in MIS-C, for example in the systemic inflammatory response syndrome (SIRS), there is an over-performance of cellular immune response, similar to what happens In patients with MIS-C, likewise, the participation of EO in SIRS is well known, reports report that in patients with a diagnosis of SIRS there are increases in lipoperoxidation, as well as decreased antioxidant capacity compared to patients without SIRS (77, 78). Likewise, the use of antioxidant nutrients has demonstrated clinical and n SIRS (79).

Similar to SIRS, there is evidence of OS involvement in KD, Some OS biomarkers, such as reactive oxygen metabolites (ROM), were increased in patients with KD naïve to treatment and favorably decreased in cases responding to treatment, in contrast to non-responding patients (80). It has also been described that there is a late increase in plasma levels of malondialdehyde and hydroperoxide after acute disease in patients with KD (81).

On the other hand, antioxidant markers are decreased in patients with KD in acute stages, such as the antioxidant power (82). Also, there is evidence of an association between the presence of manganese superoxide dismutase (*MnSOD*) rs5746136 polymorphism and susceptibility to KD (83). Other studies have proposed the use of antioxidant substances as adjuvant therapy for these patients; in particular, berberine exerts a protective effect against KD-induced damage of human coronary artery endothelial cells through the decrease of OS markers (84).

Although KD, SIRS, TSS, and MIS-C have differences, these diseases can provide a model for studying the pathogenesis of MIS-C, especially since KD, TSS, and SIRS are believed to be triggered by viral infections, sepsis, and can evolve to systemic inflammation and vasculitis (85).

## Conclusions

The disorder MIS-C is a systemic hyper-inflammation developed by some children after SARS-CoV-2 infection. It shares clinical features and molecular mechanisms with other pathological entities, such as KD. Like KD, the exacerbated immune response in MIS-C could be associated with OS development *via* NF-κB, Nrf2, endothelin-1, and others pathways. However, more studies are required to clarify the participation of OS in the pathogenesis of MIS-C.

## Further Research

Further research is needed to support confirming whether OS plays an important role in the physiopathology of MIS-C, studies should be established focused on the search for new specific biomarkers on OS in these patients.

## Author Contributions

Conceptualization of the study, OG-M and SS-F. Literature search, GV-R, IL-P, and JM-B. Writing—original draft preparation, GV-R, IL-P, OG-M, and SS-F. Writing—review and editing, JM-B, OG-M, and SS-F. All authors contributed to the article and approved the submitted version.

## Conflict of Interest

The authors declare that the research was conducted in the absence of any commercial or financial relationships that could be construed as a potential conflict of interest.

## Publisher’s Note

All claims expressed in this article are solely those of the authors and do not necessarily represent those of their affiliated organizations, or those of the publisher, the editors and the reviewers. Any product that may be evaluated in this article, or claim that may be made by its manufacturer, is not guaranteed or endorsed by the publisher.
